# Diagnostic accuracy of screening algorithms to identify persons with active pulmonary tuberculosis at prison entry: protocol of a systematic review and network meta-analysis

**DOI:** 10.25122/jml-2022-0164

**Published:** 2022-12

**Authors:** Stephanie Pape, Kabiru Gulma, Siddharudha Shivalli, Laurent Cleenewerck de Kiev

**Affiliations:** 1 Department of Global Health, Euclid University, Bangui, Central African Republic; 2 Center for Evaluation, London School of Hygiene & Tropical Medicine, London, United Kingdom

**Keywords:** tuberculosis, prison, entry screening, active case finding, 95% CI – 95% confidence intervals, AFB – Acid-fast bacilli, AUC – Area under the curve, BAL – Bronchoalveolar lavage, BCG – Bacille Calmette Guerin, CD4 – Cluster of differentiation 4, CT – Computed tomography, CXR – Chest radiography, DNA – Deoxyribonucleic acid, DOTS – Directly observed therapy strategy, ECDC – European Centre for Disease Prevention and Control, EPTB – Extrapulmonary tuberculosis, FL LPA – First-line line probe assay, FM – Fluorescence microscopy, FN – False-negative, FP – False-positive, FPR – False-positive rate, HBC – High burden country, HSROC – Hierarchical summary receiver operating characteristic, HIV – Human immunodeficiency virus, IGRA – Interferon-gamma release assay, LAM – Lipoarabinomannan, LJ – Löwenstein-Jensen, LPA – Line probe assay, LTBI – Latent tuberculosis infection, MDR TB – Multidrug-resistant tuberculosis, MGIT – Mycobacterial growth inhibitor tubes, MMR – Mass miniature radiography, MRI – Magnetic resonance imaging, NAAT – Nucleic acid amplification test, NMA – Network meta-analysis, PCR – Polymerase chain reaction, PICOS – Population, Intervention, Comparison, Outcomes, Setting, PRISMA – Preferred Reporting Items for Systematic Reviews and Meta-Analyses, PROSPERO – International Prospective Register of Systematic Reviews, PTB – Pulmonary TB, QUADAS-2 – Quality Assessment of Diagnostic Accuracy Studies (revised tool), ROC – Receiver Operating Characteristic, SD – Standard deviation, SDGs – Sustainable Development Goals, SL LPA – Second-line line probe assay, Sn – Sensitivity, Sp – Specificity, SROC – Sum ROC, STD – Sexually transmitted diseases, TB – Tuberculosis, TB-LAMP – Loop-mediated isothermal amplification, TN – True-negative, TNF – Tumor necrosis factor, TP – True-positive, TPR – True-positive rate, TST – Tuberculin skin test (Mendel-Mantoux-Test), UV – Ultraviolet, WHO – World Health Organization, ZN – Ziehl-Neelsen

## Abstract

Prison inmates are a high-risk group for tuberculosis (TB) infection and disease due to the increasing number of vulnerable fringe groups, risk factors (*e.g*., alcohol and drug addictions), contagious diseases (HIV, hepatitis), and their high-risk behavior. Compared to the general population, TB incidence and prevalence rates are significantly higher among prison inmates. Early identification of potentially infectious pulmonary TB (PTB) and targeted care of sick inmates are essential to effectively control TB within the prison system. The WHO recommends combining active and passive case-finding in prisons. No study has been published comparing the broad spectrum of screening tools using a diagnostic accuracy network meta-analysis (NMA). We aim to identify the most accurate TB case-finding algorithm at prison entry that is feasible in resource-limited prisons of high-burden TB countries and ensures continuous comprehensive TB detection services in such settings. Evidence generated by this NMA can provide important decision support in selecting the most (cost-) effective algorithms for screening methods for resource-limited settings in the short, medium, and long terms.

## INTRODUCTION

Tuberculosis (TB) continues to be one of the leading causes of death globally despite significant progress in reducing TB mortality [1]. Compared to the general population, TB incidence and prevalence are significantly higher among prison inmates due to risk factors (*e.g*., alcohol and drug addictions), contagious diseases (HIV, hepatitis), their predominantly high-risk behavior, and TB transmission favoring environment in prisons [2–4]. The World Health Organization (WHO) recommends combining active and passive case-finding in prisons [5].

Many researchers have investigated the diagnostic accuracy of different screening tests or algorithms in the inmate population [2, 3, 6–11]. Nevertheless, the studies published so far compared a limited number of tests or algorithms directly, but not all TB screening tools investigated so far applied direct and indirect comparisons, as shown in PubMed literature searches on July 3, 2020, and February 18, 2021(results not shown).

Economic evaluations about the cost-effectiveness of TB detection in prison settings often rely on relevant data like TB prevalence, transmission models, and diagnostic strategies used but regularly do not take into account the diagnostic accuracy of the screening tools and their systemic applicability in low-resource settings as some instances from literature illustrate [12–14].

By conducting a systematic review and network meta-analysis (NMA), we want to identify the most accurate TB case-finding algorithm at prison entry that is feasible in resource-limited prisons of high-burden TB countries and ensure continuous comprehensive TB detection services in such settings.

### Case finding in prisons

Generally, TB case finding in prisons comprises passive and active activities based on three strategies: self-referral, screening at prison entry, and active case-finding. Active case-finding can be set up as mandatory or voluntary testing [15]. Self-referral belongs to passive case-finding as it is on the inmates to contact internal health personnel for symptom reporting and a physical examination. This strategy often lacks success due to inmates' poor education and awareness concerning TB symptoms or prisoners' society structures [5].

According to revised European Prison Rules, prisons should establish a standard medical screening for TB encompassing other ill conditions for every inmate at entry, as prisons are responsible for caring for inmates' health [5]. As inmates, visitors, or even personnel might be latent tuberculosis infection (LTBI) carriers, and false-negative entry screening results can never be ruled out, the second pillar of active case-finding measures is screening all inmates systematically within predefined intervals. This effort aims to reduce the transmission risk among prisoners, visitors, and personnel. Finally, the entire population's TB screening frequency is a case-to-case decision based on disease prevalence, inmates' health status, and financial and personnel constraints. Screening should be conducted in prion settings with high TB prevalence in half-year intervals. Beyond these activities, active case-finding before an inmate's release is also essential to lower the transmission into the community. Methods usually applied for screening purposes include tuberculin skin testing (TST), questionnaires, and radiography; a sputum examination should follow.

For systematic TB screening, WHO experts recommend applying a screening algorithm comprising at least one screening test supplemented by at least one diagnostic test [16]. The WHO guidelines encompass ten different testing schemes combining screening and diagnostic tests for a predefined population to be screened (Table 1).

**Table 1:** Screening and diagnostic algorithms applicable for active PTB [16].

Screening and diagnostic algorithms applicable for active PTB	
**Algorithm 1 – Cough screening**	
**a**.	Followed by sputum smear microscopy
**b**.	Followed by GeneXpert
**c**.	Followed by chest X-ray (CXR) followed by sputum smear microscopy
**d**.	Followed by chest X-ray (CXR) followed by GeneXpert
**Algorithm 2 – Any TB symptom screening**	
**a**.	Followed by sputum smear microscopy
**b**.	Followed by GeneXpert
**c**.	Followed by chest X-ray (CXR) followed by sputum smear microscopy
**d**.	Followed by chest X-ray (CXR) followed by GeneXpert
**Algorithm 3 – Chest X-ray (CXR)**	
**a**.	Followed by sputum smear microscopy
**b**.	Followed by GeneXpert

CXR – Chest radiography; PTB – Pulmonary tuberculosis; TB – Tuberculosis.

Undoubtedly, all ten options proposed by the WHO are sequential algorithms. However, new technological achievements provide smear microscopy replacement and more test-combining options. The following tests, examinations, or procedures are established so far and recommended by WHO for active case-finding purposes and the subsequent diagnosis of PTB (Table 2) [16–22].

**Table 2:** Overview of screening and diagnostic tests for active pulmonary TB diagnosis.

Category	Test	Features	Pros (+)	Cons (-)	Algorithm
Screening for active TB	TB symptom screening	Questionnaire-based screening for one or more symptoms typical for PTB (incl. fever, productive or persistent cough, fatigue, night sweats, weight loss, and hemoptysis)	Assessment by non-medical personnel or medical personnel other than physicians; inexpensive method	Only for pulmonary TB (PTB) assessed; might miss cases in the absence of symptoms	Operated alone or combined, either sequentially or simultaneously: 1) sequential testing: symptom screening, followed by CXR if symptoms are reported. Usually, referral to confirmatory testing even if only symptoms screening is positive; 2) parallel testing: both tests applied must be positive to initiate further diagnostic examination
	Chest radiography (CXR)	Posterior-anterior CXR recording using conventional CXR, digital, or mass miniature radiography (MMR); Classification: any abnormality vs. normal	Independence of personal cooperation and information compared to sputum- or solely symptom-based screening	Abnormal CXR interpretation: positive screen due to abnormalities suggestive of TB, usually done by radiology or pulmonology experts; other medical personnel only distinguishing normal vs. abnormal; expensive method	
Screening of LTBI and active TB	Tuberculin skin test (TST, Mendel-Mantoux-Test)	Intradermal injection of a standardized amount of mycobacterial cell wall proteins (antigens); palpable skin thickening at the injection site within three days (immune response in infection cases)	Established positivity criteria of induration extent concerning risk groups and their risk factors	No differentiation of LTBI and active TB; always further testing needed to identify persons with active PTB; possible cross-reactions (false-positive results) if vaccinated (Bacille Calmette Guerin (BCG) vaccine) or exposed to non-TB mycobacteria; potentially false-negative test result for infection less than eight weeks ago, congenital or acquired immunodeficiency, or severe courses, e.g., in miliary TB	Applied if distinguished screening of LTBI and active TB not required; both tests can be integrated in screening algorithms focusing on active TB
	Interferon-gamma release assays (IGRAs)	Detection of increased interferon-gamma release from T-lymphocytes (immune reaction to M. tuberculosis bacterial antigens)	Low cross-reactions probability	No differentiation between LTBI and active TB; always further testing needed to identify persons with active PTB	
Diagnostic	1. Real-time polymerase chain reaction (PCR) assays/Nucleic acid amplification tests (NAAT) Examples: Xpert MTB/RIF® (Ultra) (cartridge-based), Truenat™ (chip-based)	Detection of M. tuberculosis DNA and gene mutations associated with rifampicin resistance based on enzymatic polymerization reaction (real-time PCR)	Cartridge-based assay: automatic-step, closed testing unit; infection-protective due to biocidal sample reagent; results within 2 hours; low technical complexity; next generation (Ultra assay) with significantly improved TB diagnosis sensitivity and accuracy for rifampicin resistance detection; Chip-based assay: DNA detection and rifampicin resistance test by real-time micro-PCR in automated, battery-powered devices; extraction, amplification, and detection of specific gene mutations with minimal user intervention within 1 hour; use in basic laboratories with less trained personnel; suitable for mobile on-site operations	Cartridge-based assay: specialized laboratories with appropriately trained personnel needed; uninterrupted, stable power supply, temperature control, and annual calibration required	Molecular assays intended as initial PTB diagnostic tests following screening tests
	2a. Line probe assays (LPAs) for the detection of resistance to first-line antituberculosis drugs Examples: GenoType® MTBDRplus v1 and v2, Genoscholar™ NTM+MDRTB II	Detection of M. tuberculosis and strains resistant to isoniazid and rifampicin	Highly sensitive in detecting TB in smear-positive sputum culture; beneficial for designing calculated anti-TB drug therapy concerning extent of isoniazid and rifampicin resistance (high- vs. low-dose drug resistance gens)	Less efficient in smear-negative and culture-positive samples; advanced laboratories needed	Molecular assays intended as initial PTB diagnostic tests following screening tests
	2b. Line probe assays (LPAs) for detection of resistance to second-line antituberculosis drugs Examples: GenoType® MTBDRsl	Identification of gene mutations associated with fluoroquinolone (FQ) resistance	Resistance investigation to second-line anti-TB drugs	Limited sensitivity; combination with other drug sensibility testing methods for complete drug resistance pattern determination; adequate laboratory infrastructure required due to complex analytical procedure	Not appropriate for initial diagnostic testing; not recommended to replace sputum microscopy
	3. Loop-mediated isothermal amplification (TB-LAMP) Examples: Loopamp^TM^ Mycobacterium tuberculosis complex (MTBC) detection kit	Loop-mediated isothermal amplification of target DNA at constant temperature	Low complexity, suitable for basic laboratories; visual evaluation in the reaction vessel using UV light	No drug resistance testing	Molecular assays intended as initial PTB diagnostic tests following screening tests
Diagnostic	4. Antigen detection in a lateral flow format (biomarker-based detection) Examples: Alere Determine™ TB LAM Ag	Detection of lipoarabinomannan (LAM) antigen (major bacterial cell wall component, virulence factor indicating bacteria's metabolisms or degradation) in urine	Higher sensitivity concerning TB diagnosis in HIV co-infected persons, particularly those with low CD4 cell counts, when compared to other diagnostic approaches	Less sensitive in the general population; lower diagnostic accuracy than the GeneXpert technology	Molecular assays intended as initial PTB diagnostic tests following screening tests; combination with further confirmatory diagnostic tests (e.g., PCR assays, culture, phenotypic drug sensibility testing, or line probe assays)

BCG – Bacille Calmette Guerin; CXR – Chest radiography; CD4 – Cluster of differentiation 4; DNA – Deoxyribonucleic acid; FQ – Fluoroquinolone; IGRAs – Interferon-gamma release assays; LAM – Lipoarabinomannan; LPAs – Line probe assays; LTBI – Latent tuberculosis infection; MMR – Mass miniature radiography; MTBC – Mycobacterium tuberculosis complex; NAAT – Nucleic acid amplification tests; PCR – Polymerase chain reaction; PTB – Pulmonary tuberculosis; TB – Tuberculosis; TB-LAMP – Loop-mediated isothermal amplification; TST – Tuberculin skin test; UV – Ultraviolet.

### Clinical pathway

Following the WHO recommendations, every newly admitted inmate should be screened for infectious TB before entering prison. As long as an inmate has not been tested to be unlikely to have active PTB, that person must be kept separated from peer inmates and the general facility personnel [5]. Within the admission context, applying a TB screening tool is the first step of an algorithm to identify persons with active PTB by subsequent diagnostic testing. Persons tested positively by the screening test (true and false-positives) will undergo confirmatory testing. Like the true-positives, false-positive persons not confirmed by a diagnostic test might be set on empire-driven drug therapy, a highly critical strategy due to the rising numbers and extent of MDR TB.

In contrast, persons screened negative usually will not be referred for further TB testing. That encompasses persons correctly identified as negative, meaning not having active PTB (true-negatives, TN), and persons with active PTB, *i.e*., falsely screened negative (false-negatives, FN). In the close-distance prison environment, the latter groups reflect a serious risk for TB transfer in the facilities and disease transmission to peer inmates, personnel, and visitors, particularly if the affected persons are asymptomatic or do not report symptoms voluntarily as intended in the passive case-finding approach [19].

Confirmatory tests usually applied in prison settings are sputum examinations, including sputum smear microscopy, WHO-approved NAAT, *e.g*., GeneXpert MTB/RIF, and bacterial culture of *M. tuberculosis* [5, 19]. These tests may also serve as reference standards in prison settings, with bacterial culture still being the gold standard of TB detection (Table 3) [19, 23].

**Table 3:** Reference tests for TB diagnosis usually applied in prison settings.

Test	Features	Pros (+)	Cons (-)	Diagnostic Value
Mycobacterial culture	Confirmation of M. tuberculosis in cultured sputum (solid [Löwenstein-Jensen (LJ)] or liquid (mycobacterial growth inhibitor tubes (MGIT culture) medium)	Differentiation of M. tuberculosis from non-tuberculous bacteria; MGIT culture more sensitive due to automated reading techniques; LJ applicable in resource-limited settings; MGIT results after 10–14 days	High false-negative rate in immuno-compromised individuals; MGIT culture more expensive than LJ; LJ results after 8 weeks; MGIT with decreased specificity due to extended contamination	Gold standard of active PTB diagnosis
Sputum smear microscopy	Detection of acid-fast bacilli (AFB) in sputum [original Ziehl-Neelsen method (ZN), more advanced technologies like auramine-stained fluorescence microscopy (FM)]	Widespread availability; FM with 10% increased sensitivity compared to the ZN method, but lower specificity; further improved sensitivity and specificity by physico-chemical sputum processing	Risk of false positivity due to artifacts and its inability to differentiate between M. tuberculosis and other AFB	Essential tool for TB diagnosis; 3 sputum samples (5–10 ml, lower limit: 3 ml) recommended for better specificity, incl. at least 1 early-morning specimen, followed by 2 others after not less than 8 hours
Nucleic acid amplification tests (NAAT)	Identification of bacterial particles by DNA-based molecular techniques	Higher sensitivity of M. tuberculosis detection compared to microscopy; significant time advantage over sputum culture; results after only a few hours; TB diagnosis of immuno-compromised individuals; screening of bacterial strains for antibiotic resistance; support of immediate use of calculated antibiotic therapy	PTB cannot be excluded based on a negative NAAT	Single positive sputum sample sufficient for diagnosing active PTB, even in a negative AFB smear with moderate to high suspicion of active TB

AFB – Acid-fast bacilli; DNA – Deoxyribonucleic acid; FM – Fluorescence microscopy; LJ – Löwenstein-Jensen; MGIT – Mycobacterial growth inhibitor tubes; ml – Milliliter; NAAT – Nucleic acid amplification tests; PTB – Pulmonary tuberculosis; TB – Tuberculosis; ZN – Ziehl-Neelsen.

Other screening and diagnostic tests than those mentioned are initially not classified as clinically relevant for the review and the planned network meta-analysis. Experts do not consider them among the standard screening and diagnostic tests for active PTB currently widely established in practice. However, the search strategy will not be narrowed down to specific technologies. Should the review identify other technologies that could play a role in screening and diagnosing active PTB, the respective screening or diagnostic tests would be included. Thus, a reevaluation of the screening and diagnostic tests, examinations, and procedures to be included will be performed based on the review's first-round results.

### Why this review?

In prison settings, active PTB at entry screening is optimally provided to any newly admitted person [6]. However, in the real world, screening may only occur for certain target groups, if necessary, or in a limited setting for other reasons. Currently, there is no data available concerning the most accurate TB case-finding algorithm at prison entry that is feasible in resource-limited prisons of high-burden TB countries and ensures continuous comprehensive TB detection services in such settings. We aim to identify a screening strategy or tool that fulfills all the before-mentioned conditions. By conducting a systematic review of the evidence available regarding the diagnostic accuracy of active PTB screening algorithms at prison entry, we want to provide evidence to that issue. Our work started immediately after publishing the review protocol on PROSPERO [24]. Studies identified as eligible will subsequently be used for an NMA.

### Objectives

This systematic review and network meta-analysis aims to investigate the diagnostic accuracy of active PTB screening tests and algorithms, *i.e*., the combination of screening and diagnostic tests applied at prison entry to newly admitted inmates.

Additionally, we plan to research heterogeneity concerning study population and individual participant characteristics, screening tests used and definition contingency, reference standards used, WHO region, country income group, and study's representativeness concerning the screening practice determined. If we get enough eligible studies, we will do separate meta-analyses for the individual screening and diagnostic tools in advance of the network meta-analysis to assess the evidence-based diagnostic validity for each test, examination, or procedure separately.

Besides researching which diagnostic screening algorithm for prison entry is the most accurate in any prison setting, we want to explore any differences in entry screening algorithm applications between prison settings in TB high-burden countries compared to prison settings in countries not announced as TB high-burden countries. We assume that prisons located in countries listed on WHO's former and current TB high burden country (HBC) lists operate with limited structural, financial, and personnel resources [25].

## MATERIAL AND METHODS

### Criteria for consideration of studies in systematic review/network meta-analysis

The content of Table 4, also applied by ECDC colleagues researching prison settings [15], provides an overview of the key characteristics addressed in this review using the PICOS algorithm, followed by explaining the essential criteria crucial for study inclusion.

**Table 4:** Screening for active PTB at prison entry – PICOS algorithm [15].

Screening for active PTB at prison entry	
**P**	Newly arriving inmates of any age at entry in prison settings
**I**	Active pulmonary TB case-finding by a screening algorithm
**C**	A composite reference standard comprising bacteriological confirmation by solid/liquid culture, and/or positive sputum smear(s), and/or a WHO-endorsed nucleic acid amplification test (NAAT), e.g., GeneXpert MTB/RIF
**O**	Diagnostic accuracy data, such as sensitivity, specificity, true-positive values, false-positive values, true-negative values, false-negative values
**S**	Prisons, jails, and other custodial settings functioning as a prison (excluding migrant centers and police detention rooms)

NAAT – Nucleic acid amplification test; PTB – Pulmonary tuberculosis; TB – Tuberculosis; WHO – World Health Organization.

### Study type

Only studies in which the index test and the reference standard intended for screening and diagnosis were applied in a restricted time window are eligible for the subsequent network meta-analysis. Given the expected limited number of publications dealing with the review topic, we will initially accept various study types for review inclusion if the study's objective and data provided matches our aim sufficiently. These study types comprise cross-sectional, case-control, cohort studies, and experimental studies with or without randomization. Regarding limitations unique to the distinguished study designs, we will investigate and evaluate the resulting biases and other methodological issues within the methodological quality assessment. We will exclude those from our further analyses if studies are assessed as ineligible due to methodological concerns. To be included, studies must deliver data, *i.e*., TP, FP, TN, and FN values that allow 2x2 contingency table calculations for an index test compared with the reference standard.

Furthermore, studies must report a value of identified TB cases greater than zero. Additionally, cases must be detected at the time point when entry screening is performed. Later occurring incident cases will not be recognized for inclusion and data analyses. Consideration should also be given to whether meta-analyses of diagnostic studies are eligible for inclusion in network meta-analysis.

Van't Hoog and colleagues used a time limitation from 1992 onwards to argue with the directly observed therapy strategy (DOTS) implementation and subsequent significant changes in treatment standardization and passive case finding [19]. We will not apply any time limits in our review. However, if we include studies conducted before 1992, we will investigate the heterogeneity of that period threshold.

### Participants

Eligible participants are persons newly admitted to any custody facility encompassed by the term prison. Inmates are a high-risk population for TB, HIV, hepatitis, other infectious diseases, and risk behavior; they are also hard to reach for health interventions [5, 26]. Thus, their health status is usually unknown when admitted to prison for the first time. Regarding former inmates, even if the medical records exist, those may not be available just in time to deliver supportive information for the entry screening. Conclusively, the individual's status of active PTB is commonly unknown. The same is true for HIV and other communicable and non-communicable diseases.

Generally, the majority of inmates are males who are rarely younger than 15 years [27]. However, information about females and children under 15 will also be recognized for inclusion if adequately reported within the studies. Newly admitted inmates may originate from the country the study is conducted within or may immigrate to that country for any reason and from any foreign country. We will exclude studies that present participants that differ significantly from those described before.

Besides that, we will not include studies exclusively reporting passive case finding or active case finding later than prison entry. If studies provide data for persons screened while under TB treatment, we will remove those individuals' data from our analyses to reduce bias.

### Target conditions

The target disease is active PTB, which is infectious due to *M. tuberculosis* bacilli in a person's sputum. Carrying out TB screening at prison entry for each new inmate is likely to detect asymptomatic persons or persons without CXR abnormalities depending on the screening tool applied and whether such persons would be referred to sputum examination for whatever medical reasons.

Data analysis in the Berlin prison system, Germany, found that 25% of PTB patients were asymptomatic at diagnosis [28]. Therefore, we will include studies that included asymptomatic or CXR-negative persons diagnosed as active TB cases based on index test(s) other than symptoms or CXR. We will particularly focus on those studies in the methodological quality assessment. As latent TB infection (LTBI is not targeted by prison entry TB screening, we will exclude those studies that exclusively investigate LTBI conditions.

EPTB, also a form of active TB, is not focused on in this review due to its limitation on entry screening. Culture-negative active PTB is usually diagnosed based on clinical symptoms and CXR findings highly suggestive of TB if not caused by other reasonably likely conditions. For culture-negative patients, empirical drug therapy may be initiated based on clinical diagnosis in resource-limited settings where bacterial confirmation by culture is not a routine laboratory procedure. We will follow van't Hoog and colleagues' approach and will refrain applying clinical algorithms as a reference standard for this review [19]. Given the risk of infectivity, persons with EPTB or culture-negative active PTB are of minor concern in prison entry screening compared to culture-positivity or even positive sputum smear microscopy. Due to WHO's standard-setting role, we will restrict our reference standard definition to those tests officially endorsed by WHO for TB screening or diagnosing purposes, as van't Hoog and colleagues stated [19].

### Index tests

- Screening for TB symptoms;
- Chest X-ray (CXR) screening;
- Tuberculin skin test (TST, Mendel-Mantoux-Test);
- Interferon-gamma release assays (IGRAs);
- Real-time polymerase chain reaction (PCR) assays/Nucleic acid amplification tests (NAAT);
- Line probe assays for the detection of resistance to first-line antituberculosis drugs (FL LPA);
- Line probe assays for the detection of resistance to second-line antituberculosis drugs (SL LPA);
- Loop-mediated isothermal amplification (TB-LAMP);
- Antigen detection in a lateral flow format (biomarker-based detection) (TB LAM).

### Reference standards

The gold standard for definitive diagnosis of active PTB is the confirmation of *M. tuberculosis* in cultured sputum collected on three different days, with liquid culture being more sensitive than solid culture [19]. We will adopt the reference standards used by van't Hoog and colleagues for our analyses: bacteriological confirmation by solid/liquid culture and/or positive sputum smear(s) and/or NAAT [19]. Studies applying any of these combinations will be accepted for inclusion. However, we will also check studies' eligibility for inclusion that apply only one component of the composite reference standard. Suppose we should identify studies probably eligible for our review that used a reference standard other than that defined here. In that case, we will critically assess them for their methodological quality in each domain of the QUADAS-2 tool, particularly concerning the reference standard domain [29]. Besides, we will separately research the effect of different reference standards within the heterogeneity analyses.

### Types of outcome measures

#### Main outcome(s)

Studies must deliver data, such as sensitivity, specificity, true-positive values, false-positive values, true-negative values, and false-negative values, that allow 2x2 contingency table calculations for an index test compared with the reference standard. Furthermore, they must report a value of identified TB cases greater than zero. Additionally, cases must be detected at the time point when entry screening is performed.

#### Additional outcome(s)

Area under the curve (AUC).

### Search strategy for the identification of studies

A robust search strategy will be used to identify all relevant studies reporting data on the accuracy of screening tests and screening algorithms for active PTB. Therefore, no restrictions will apply concerning the reference standard or screening test used.

#### Search in electronic media

We will conduct literature searches in PubMed, Global Index Medicus, and the Cochrane Library using the search strategies listed in Appendix 1. SP will perform the initial searches. Time and language restrictions are waived, and if a translation of non-English articles becomes necessary, a feasible solution will be sought. There is no restriction on regions and countries. We aim to gather all existing evidence for TB screening at prison entry. Any separation regarding low-resource settings will be done within the data analyses.

#### Search in other sources

We expect grey literature sources to provide useful evidence not published in peer-reviewed journals. For that reason, we are going to collect appropriate data from articles, conference abstracts, research reports, study or other protocols, guidelines, or other documents by Google and Google Scholar searches and from the following websites visited by the ECDC working group for their review about TB in prisons [15].

Regarding grey literature search terms, depending on the search engines or websites, the search strings used for the peer-reviewed articles search will be used and adapted if necessary. On prison-specific websites, the prison search string will be left out. All searches will be documented in detail (data not shown).

#### Further steps

It is also planned to search the reference lists of all publications considered for this review for further studies and reviews in this area and repeat the process until no additional new titles are found [30].

### Data collection and analysis

#### Study selection

One reviewer (SP) will merge the references obtained from the literature sources searched, followed by deduplication. Two reviewers (SP and KG) will check the remaining references for relevance by titles and abstracts and, later, by full texts independently. Suitable studies are extracted. All results from the first and full-text screening are compared, and different assessments regarding the inclusion or exclusion of studies are discussed. Table 5 showcases the inclusion and exclusion criteria.

**Table 5:** Inclusion and exclusion criteria [15].

	Inclusion	Exclusion
Study design	Meta-analysis or systematic review, in total or single studies of those; Randomized controlled trials (RCTs); Non-randomized, prospective comparative studies; Observational studies (e.g., cohort studies, case-control studies); Cross-sectional studies; Diagnostic studies.	Narrative reviews; Case reports/case series; Non-pertinent publication types (e.g., expert opinions, letters to the editor, editorials, comments, conference abstract/poster, news, consensus documents, chapter); Animal studies; Genetic studies, biochemistry, or molecular studies; Modeling studies; Outbreak studies.
Study characteristics	Study duration (not limited); Number of subjects (not limited).	Concerns about methodological quality (inherent methodology or insufficient methodology information).
Study population	Persons in prisons, jails, and other settings that function as a prison; Detained persons, including persons in remand.	Persons in police custody; Persons in migrant detention centers; Facility personnel; Any kind of visitors.
Outcomes	Quantitative data applicable for diagnostic accuracy calculations.	Lacking data applicable for diagnostic accuracy calculations.

If both reviewers fail to reach a consensus in this process, the disputed publications will be submitted to a third reviewer (SS) for decision. The references excluded during full-text screening will be listed in the systematic review stating the reasons for exclusion according to Table 6.

**Table 6:** Exclusion criteria for studies in the systematic review – PRISMA flow chart.

Exclusion criteria	
**E-0**	Duplicate
**E-1**	Incorrect setting
**E-2**	No prison entry screening
**E-3**	Not screened for active PTB
**E-4**	Study design (e.g., comment, letter, editorial, narrative review, case report, case series)
**E-5**	Insufficient information about study methodology
**E-6**	Insufficient information about screening procedure (tests used, simultaneous/sequential testing etc)
**E-7**	Insufficient information about the study population
**E-8**	No data about diagnostic accuracy measures (sensitivity, specificity, true-positives, false-positives, true-negatives, false-negatives)

PTB – Pulmonary tuberculosis.

If a study can provide useful data but is not presented in the publication, contact is made with the authors to obtain the results required. If similar data are found in multiple publications that are not mere duplicates, the publication that includes the largest number of patients or the most meaningful or comprehensive information will be included in the review.

All searches and further process steps will be documented in detail. The study selection process will be showcased using the PRISMA statement flow diagram, generally accepted and used for systematic reviews and meta-analyses [31].

#### Data extraction and management

Regarding data extraction for journal articles, the following data will be extracted from the included studies into a pre-standardized Excel-based data matrix, where the publications will be entered row by row via an identification number (ID-No.; to guarantee a secure identification) and the relevant data column by column [15]:

- Study ID-No;
- Bibliographic data of the (primary) publication: author, title, year of publication, journal, country, study design;
- Study characteristics: study objective, prison setting, design, period/duration, follow-up;
- Basic, clinical and demographic information of the study population: prison setting (prison, jail etc), population description, number of subjects/patients, inclusion and exclusion criteria, age, sex, health-related information (other comorbidities, nutrition status, risk factors, *e.g*., drug addiction, alcohol abuse, smoking), previous history of TB, previous incarcerations;
- Data source description and relevant definitions;
- Screening test details: testing method (CXR, TST etc), test application sequence (simultaneous, subsequent etc), test offer (mandatory, opt-in, opt-out), screening extent (every new inmate, high-risk persons), timing, consent;
- Criteria to define positive and negative test results;
- Assay type;
- Outcome results: Number of true-positives, false-positives, true-negatives, and false-negatives as published or calculated based on the respective information given in the publication;
- If available, the area under the Receiver Operating Characteristic (ROC) curve;
- Reference standard: definition of active PTB, time used between reference standards and index tests;
- Drop-outs: missing data due to several participants for whom index test or reference standard results were missing after they were recruited to the study.

Regarding data extraction for grey literature documents and websites, grey literature findings will be operated similarly to journal articles. Still, they will be marked as grey literature documents and adapted regarding reference, source, and document type.

#### Assessment of methodological quality

Using the Quality Assessment of Diagnostic Accuracy Studies-2 (QUADAS-2) tool, two reviewers (SP and KG) will independently assess each included study's methodological quality [29]. The Cochrane Collaboration also recommends this. The tool consists of four domains:

- Patient selection;
- Index test;
- Reference standard;
- Patient flow;

Each domain is assessed for bias risk, and the first three domains are additionally assessed for applicability. We do not expect a need for incorporation-bias risk assessment as long as sputum smear microscopy or a NAAT is used not as an index test. Given the duration until culture positivity, it is unlikely to identify studies applying bacterial confirmation by culture as an index test.

The components of each of these domains and a rubric detailing how to operationalize bias risk assessment are provided in Appendix 2. Each item's classification depends on the signaling question's topic adequately responded to by the study authors. The categories are: (1) yes, meaning adequately responded, which is equal to a low risk of bias, (2) no, meaning inadequately responded, which is equal to a high risk of bias, (3) unclear, meaning data being insufficient, which is equal to an unclear risk of bias. However, the QUADAS-2 data will not be used to calculate an overall quality score. A narrative review will describe the number of studies classified as having a high, low, or unclear risk of bias and applicability assessments.

With respect to determining the degree of agreement or inter-rater bias, the first piloting of the QUADAS-2 tool is performed using two studies for independent evaluation by the same reviewers conducting the quality assessment [29]. If the agreement is low, we will revise the signal questions. Regarding the main quality assessment, both reviewers will discuss differing results. In cases where they cannot achieve consensus, a third reviewer (SS) will resolve the issue.

#### Statistical analysis and data synthesis

First, we will describe all studies identified for review inclusion by the most relevant characteristics determined in the data extraction and management section. Regarding screening definition requirements, we will determine appropriate categories depending on the single index tests used in the screening algorithms as screening or confirmatory tests combined with the individual test characteristics and the diagnostic conclusions derived from them.

Given the fact that a significant number of studies probably eligible for review inclusion were conducted in resource-limited settings, we will adopt the reference standard classification that van't Hoog and colleagues used for subgroup analysis in their systematic review: culture, culture and smear combined, or NAAT only, versus smear microscopy only [19]. Krippendorff's alpha calculation will be applied for a reliability investigation of the risk of bias assessment between the investigators [32].

#### Individual meta-analyses

Based on the diagnostic test accuracy framework for analyzing a single dichotomous test, each study's extracted data is transformed into a 2x2 contingency table that cross-classifies the binary test results with the binary reference standard. The resulting data from the included studies on the true-positive, false-negative, false-positive, and true-negative status are fed into the statistical software, and the estimates of sensitivity (Sn) and specificity (Sp) and their 95% confidence intervals (95% CI) are calculated. The graphical presentation of the individual study results is provided by plotting the estimated Sn and Sp in a forest plot.

If more than one algorithm were published in the primary studies, all algorithms' estimated accuracy would be examined. The Sn-Sp value pairs are transformed into a meta-analysis if sufficient data for individual screening and diagnostic tests are available. A bivariate binomial model analyzing Sn and Sp pairs jointly in each case (*e.g*., the generalized linear mixed model approach by Chu and Cole) is used, applying the *glmer* function in the R package *lme4* (version 1.1-25) [33]. So, the correlation between true-positive rate (TPR) and false-positive rate (FPR) and their between-study standard deviation (SD) can be estimated via random effects providing information on result heterogeneity [34]. If studies report multiple algorithms, the most used algorithm from all included studies is included in the meta-analysis (and, as a sensitivity analysis, all other algorithms for which a sufficient amount of data exists will feed the meta-analysis).

This approach's limitations cannot be avoided, given the lack of standard algorithms. A detailed discussion of this issue will be provided in the discussion section of the later review. Where appropriate, the sensitivity of the findings concerning the choice of algorithms will be investigated. R software will be used for the "frequentistic" approach to the meta-analyses, and Stan in conjunction with R for the Bayesian approach [35]. Details of the a priori, probabilistic, and a posteriori distribution for the analyses performed with Stan are provided [36].

Furthermore, the hierarchical summary receiver operating characteristic (HSROC) approach results are reported. If pooled studies use a common algorithm, the pooled estimates of Sn and Sp from the bivariate model are also reported. If pooled studies do not use a common algorithm, the sum ROC (sROC) for all studies across all algorithms will be determined, and the results of the HSROC model will be explored after it has been adjusted for the studies across the different algorithms. This procedure is used to explore limit effects. Model fit is determined using the likelihood ratio test for the frequentistic approach and the Bayesian methodology's deviance information criterion.

All reliable cumulative accuracy estimators' emergent implications are examined by eliciting the number of false-positives and false-negatives in populations with different prevalences.

Besides, natural frequencies are presented, and alternative measures such as likelihood ratios and predictive values are provided. Alternatively, given the pretest probability and likelihood ratios, the likelihood ratio nomogram can be used to survey the post-test probability of disease [37].

#### Network meta-analysis

Regarding the diagnostic accuracy comparison of different screening algorithms, a diagnostic NMA approach serves for evidence synthesis. The preferred method for simultaneously combining direct and indirect evidence is the beta-binomial analysis of the variance model for diagnostic test accuracy data NMA by Nyaga et al. [38].

The arm-based generalized linear mixed model handles Sn and Sp as repeated measures jointly through a copula function while assuming randomly missing tests/arms. In the Bayesian framework, the model fit is realized with beta (1,1)=U(0,1) as prior distribution on the hyper-parameters by Stan through the R package rstan (version 2.21.2) [39]. We plan the NMA repetitions for different certainty levels of TB diagnosis. If the data available for some subgroups or screening definitions are insufficient to create pooled Sn and Sp estimates, we will provide an appropriate description of our findings [19].

#### Investigation of heterogeneity

Several factors are included in the investigation of possible sources of heterogeneity [19]:

**I. Test algorithm**

- Screening test(s) used;
- Diagnostic (confirmatory) test(s) used;
- Reference standard used (culture, smear microscopy, NAAT, or as specified by study authors);
- Screening algorithm(s) used:
  1. Sequential testing:
    - One screening test followed by one diagnostic test;
    - One screening test followed by one diagnostic test followed by another diagnostic test;
    - One screening test followed by another screening test followed by one diagnostic test.
  2. Parallel testing:
    - Two simultaneous screening tests followed by one diagnostic test.
- Consistency regarding screening test definition (agreement of the predetermined testing characteristics indicating TB with the actually tested characteristics).

**II. Target disease**

- Reference standards used: Bacteriological confirmation by solid/liquid culture, and/or positive sputum smear(s), and/or GeneXpert MTB/RIF.

The clinical diagnosis is made based on the results of the before-mentioned laboratory procedures. The cause for such diagnostics is usually given by patients suffering from the following TB-typical symptoms: persistent, productive cough, night sweats, weight loss, fever, abnormal fatigue, hemoptysis, lymph node swelling, and thoracic or abdominal pain [23].

The specimen collection for testing for *M. tuberculosis* should be done before the initiation of treatment. If microscopic detection of acid-fast rods is not successful in sputum specimens collected on three different days, overt pulmonary tuberculosis can be ruled out. TB diagnosis by culture also proves infectivity but requires several weeks for positivity. Nevertheless, detection in culture is the gold standard of TB diagnosis and is also significant for resistance testing [23].

**III. Target population**

- TB prevalence in the general population (country-specific);
- TB prevalence in the prison population (by country);
- Study population's representativeness for the screening practice determined (best practice: full-scale screening, *i.e*., testing of every newly admitted person);
- WHO regions;
- Income groups (country level; according to the World Bank's classification: low-income, lower-middle-income, upper-middle-income, high-income);
- Age;
- Sex;
- First-time versus multiple detentions;
- HIV status, or if unknown on the individual level, HIV prevalence reported for the study population;
- Hepatitis status (focus on hepatitis B and C);
- Smoking status;
- Alcohol addiction;
- Drug addiction;
- Diabetes status;
- STD status (STD – sexually transmitted diseases).

**IV. Study quality (QUADAS 2)** [29]

We will investigate our results within all QUADAS-2 domains by step-wisely, excluding studies assessed as low or unclear quality.

A descriptive analysis is performed using the visual examination of the Sn and Sp forest plots and the ROC plot concerning the Seffects of heterogeneity. The visualization is done using the statistical software already applied for the other analyses.

If suitable studies are available, meta-regression (bivariate models) will be performed to investigate heterogeneity. The estimated parameters thus obtained, if appropriate, are used to map the overall ROC plot, including the collected ROC curves, point estimates, confidence, and prediction intervals [19].

Where appropriate, we will also examine differences in diagnostic accuracy in subgroups. However, these subgroup analyses would be purely exploratory and reported with appropriate caution regarding their informative value.

#### Sensitivity analyses

If full-scope sensitivity analyses were not already performed as part of the heterogeneity investigation, an additional sensitivity analysis is performed on other study quality aspects. Furthermore, the effects of studies in which particular features may affect our accuracy results will be evaluated by excluding them.

#### Evaluation of reporting bias

Regarding reporting or publication bias investigation, funnel plots will be created for each screening test or test algorithm. Although diagnostic studies are hardly regulated compared to therapeutic studies, and thus uncertainties exist in operationalizing and interpreting publication bias in diagnostic test accuracy studies, this approach is defensible in terms of scientific evidence gain.

#### Evaluation of Evidence Quality

We refrain from evaluating evidence quality.

## CONCLUSION

As diagnostic and treatment options increase, international institutions such as the (WHO), the ECDC, and the Cochrane Collaboration use NMA to synthesize evidence for guideline development and answer complex questions. Nonetheless, compared with proper pairwise meta-analyses, NMA results have lower confidence levels and should be interpreted with caution and knowledge of existing limitations. NMAs represent a significant, practical advancement of conventional pairwise meta-analyses because they allow effect estimates using direct and indirect comparisons of multiple health technologies.

For TB screening in new prison admissions, the evidence generated by NMA can provide important decision support in selecting the most (cost-) effective algorithms for screening methods to be implemented in resource-limited settings in the short, medium, and long terms.

## Supplementary Material





## Annotation

The present protocol is largely based on the following references:

- European Centre for Disease Prevention and Control. Systematic review on the diagnosis, treatment, care, and prevention of tuberculosis in prison settings. Stockholm: ECDC; 2017. Available from: https://www.ecdc.europa.eu/en/publications-data/systematic-review-diagnosis-treatment-care-and-prevention-tuberculosis-prison;
- van't Hoog AH, Langendam M, Mitchell E, Cobelens FG, Sinclair D, Leeflang MM, Lönnroth K. Symptom- and chest-radiography screening for active pulmonary tuberculosis in HIV-negative adults and adults with unknown HIV status. Cochrane Database of Systematic Reviews 2014, Issue 1. Art. No.: CD010890. 10.1002/14651858.CD010890.

## Personal thanks

We thank Vasumathi Sriganesh for her advice concerning search strategy development.
