# Supplementary material for: Diagnostic accuracy of screening algorithms to identify persons with active pulmonary tuberculosis at prison entry: protocol of a systematic review and network meta-analysis
Source: J Med Life. 2022 Dec;15(12):1464–75. doi: 10.25122/jml-2022-0164 (PMC9884352; doi:10.25122/jml-2022-0164)
Supplement: Supplementary file 1 [file JMedLife-15-1464-s001.pdf]

Appendix

Appendix 1. Study design.

Part/title	Item number	
**Title and summary**	1-a<br>1-b	Investigating the efficacy of Ginkgo biloba therapy on the cognitive function of patients undergoing treatment with electric shock: a double-blind clinical trial.<br>In this study, 200 mg capsules of Ginkgo biloba and a placebo were given to two groups of patients using a double-blind method. Then, the patient's cognitive function was evaluated by WMS-III and MMSE questionnaires, and the positive effect of the intervention was assessed.
**Introduction**<br>Background and objectives	2-a<br>2-B	The use of electroshock therapy by Ogoserleti became common in psychiatric disorders, and forgetfulness and cognitive impairment were its complications. Today, complementary and alternative medicine methods in its prevention are considered in systematic review studies. Ginkgo biloba extract improves cognitive function in patients receiving electro-shock therapy, reduces cognitive complications, and improves the quality of life. The mean scores of MMSE and WMS-III questionnaires in the intervention group are higher than the control group.
**Procedure**<br>Experimental design	3-a<br>3-b	The study was performed as a double-blind and parallel clinical trial. After dividing the subjects into two groups the intervention was performed randomly.<br>The eligibility criteria for the study did not change after the start of the study due to the absence of side effects.
Participants	4-a<br>4-b	Age 18–60, suffering from psychotic and mood disorders diagnosed after DSM5 criteria approved by two psychiatrists, in the treatment plan of 6–12 sessions of shock therapy, literate, ability to communicate verbally, no disease, mental disability, or serious cognitive impairment, inpatients or outpatients treated with electroshock referred to the psychiatric ward of Ahvaz Golestan Hospital were included in the study after ethical approval.
Intervention	5	The intervention was performed in two groups in the form of a double-blind clinical trial and was performed in psychiatry patients and outpatients receiving electroshock therapy.
Outcomes	6-a<br>6-B	The cognitive assessment scales were MMSE and WMS III, performed three times before the start of the treatment process, after receiving 4 sessions of shock therapy, and 72 hours after the last session of shock therapy.<br>Due to the absence of side effects in the course of treatment, there was no significant change in the trial outcome.
Sample size	7-a<br>7-b	Considering the reference article where the mean and standard deviation of cognitive status after electroshock therapy based on MMSE in the placebo group was equal to 26.9 and 1.28 and 28.1 and 1.28 in the treatment group, and considering alpha 0.05 and p equal to 0.1 (referring to PASS software, sample size section based on the comparison in the independent group), the sample size was equal to 35 people in each group, with a prediction of 10% drop in sample size. Finally, 40 people in each group were considered.<br>No study interruption occurred.
**Randomization**<br>Sequence generation	8-a<br>8-b	Subjects were divided into two groups of treatment and placebo equally based on the randomized block permutation method to eliminate confounders.<br>Randomization was unrestricted and was divided equally into treatment and placebo to eliminate confounders.
Assignment concealment method	9	Patients were divided into two groups with even and odd numbers, and medicine and placebo were distributed among the patients in numbered containers as blinds.
Participants run	10	The assistant identified the random assignment sequence, the psychologist registered the participants, and the nurse assigned the participants to the interventions.
Blindness	11-a<br>11-b	The patients under study, the service providers, and the people who made the analysis were blinded.<br>The blinding method was similar in studies that used ginkgo biloba for vascular dementia and Alzheimer's dementia.
Statistical method	12-a<br>12-b	For quantitative variables, mean or median was used to describe the data, and standard deviation or mid-quarter amplitude was used to describe the data scatter. For qualitative variables, frequency and percentages were used to describe the data. The normality of the data was based on the Shapiro-Wilk test and Q-Q diagram.<br>Independent t-test, Chi-square, ANOVA, Pearson correlation coefficient, as well as couple t-test and, if necessary, repeated measures test or Friedman test were used to analyze univariate data. A linear mixed model with the control of independent variables was used for multivariate data analysis. A significance level of 0.05 was considered. All tests were performed using SPSS software version 22.
**Results**<br>Participant flow	13-a<br>13-b	The number of eligible participants in the study was identified as 100 people, 13 of whom were excluded from the study (6 due to lack of entry character, 5 were reluctant to participate, and 2 changed the therapy plan). 87 people were divided into two groups: intervention (43) and placebo (44).<br>Out of 44 patients in the control group, 2 were given personal consent and discharged, and 2 did not want to continue taking the placebo and were excluded from the study. Out of 43 patients in the intervention group, one was not followed up due to discharge with personal consent, and 2 were excluded.

Appendix 1. Continued.

Part/title	Item number	
Recruitment	14-a<br>14-b	After identifying the disorder and determining the treatment plan, the questionnaires were completed 24–48 hours before the start of ECT and 24 hours after the 4th session, and 72 hours after the last shock therapy session.<br>Patients' limited hospitalization time, the limited opportunity to do the study, financing problems, and loss of patients and staff cooperating in the project were the reasons for stopping the project.
Basic information	15	The table containing demographic information and clinical characteristics of the two intervention and control groups is attached.
Number of people analyzed	16	Participants were divided into two groups: intervention (n=40) and control (n=40).
Consequences and estimate	17	To improve the cognitive function results after the intervention, we used the Cohen effect size with a 95% confidence. As a result, 2.44 for WMSIII and 1.33 for MMSE were obtained.
SUB analysis	18	WMS-III test was analyzed in 7 subgroups of general memory, temporal-spatial orientation, mental control, logical memory, repetition of numbers, visual memory, and word association in two groups of intervention and control.
Dangers	19	No risk or side effects were seen in the intervention and control groups.
**Conclusion**<br>Limitation	20	Small sample size, low financial allocation, change of treatment plan during the project, patients' dissatisfaction with entering the project and patients leaving the project, and lack of treatment were some of the problems in the research process.
Generalizability	21	Due to the lack of side effects and positive effects on cognitive function, this study can be used after ECT for patients in any place with access to ginkgo biloba compounds.
Interpretation	22	The positive results of Ginkgo biloba on the cognitive function of patients after ECT were demonstrated by MMSE and WMS-III tests without obvious adverse effects, which was consistent with similar studies in the field of efficacy in dementia and amnesia and its antioxidant effects.
**Other information**<br>Registration	23	U-98224, School of Medicine, Ahvaz Jundishapur University of Medical Sciences, Ahvaz, Iran
Protocol	24	No specific protocol has been considered, and the explanation of how to perform the intervention is clearly explained in the methods section.
Funding	25	The project was funded by the Vice Chancellor for Research of Ahwaz Jundishapur University of Medical Sciences, Iran, and no other body was involved.
