# Supplementary material for: Diagnostic accuracy of screening algorithms to identify persons with active pulmonary tuberculosis at prison entry: protocol of a systematic review and network meta-analysis
Source: J Med Life. 2022 Dec;15(12):1464–75. doi: 10.25122/jml-2022-0164 (PMC9884352; doi:10.25122/jml-2022-0164)
Supplement: Supplementary file 2 [file JMedLife-15-1464-s002.pdf]

Appendix 1. The search strategies applied in this systematic review are, in most parts, identical to the search strings used in the systematic review of the ECDC working group [15].

1) PubMed

#1 Prison settings

"Prisons"[Mesh] OR "Prisoners"[Mesh] OR prison*[tw] OR penal[tw] OR jail*[tw] OR reformator*[tw] OR custodial[tw] OR custody[tw] OR gaol*[tw] OR remand*[tw] OR penitentiar*[tw] OR detention*[tw] OR correctional[tw] OR detainee*[tw] OR inmate*[tw] OR imprison*[tw] OR confinement[tw] OR incarcerat*[tw] OR cellmate*[tw] OR lockup*[tw] OR penitentiar*[tw] OR penal institution*[tw] OR detention facilit*[tw] OR bastille*[tw] OR clink*[tw] OR dungeon*[tw] OR guardhouse*[tw] OR slammer*[tw] OR stockade*[tw] OR statesville*[tw] OR up the river[tw] OR brig*[tw] OR bullpen*[tw] OR jailhouse*[tw] OR rack*[tw] OR solitar*[tw] OR stir*[tw] OR "black hole*"[tw] OR "detention camp*"[tw] OR "house of correction"[tw]

#2 Active case finding

"Mass Screening"[Mesh] OR "Mandatory Testing"[Mesh] OR screen*[tw] OR "case finding"[tw] OR "case-finding"[tw] OR casefinding[tw] OR "cases finding"[tw] OR "case identification"[tw] OR "cases identification"[tw] OR testing[tw] OR "rapid test"[tw] OR "rapid tests"[tw] OR "Early diagnosis"[Mesh] OR early diagnos*[tw] OR early detect*[tw] OR early test*[tw] OR "clinical evaluation"[tw] OR "clinical evaluations"[tw]

#3 TB

"Tuberculosis"[Mesh] OR "Mycobacterium tuberculosis"[Mesh] OR "Mycobacterium avium"[Mesh] OR "Mycobacterium bovis"[Mesh] OR tuberc*[tw] OR "Kochs Disease"[tw] OR "Koch's Disease"[tw] OR "Koch Disease"[tw] OR TB[tw] OR LTB[tw] OR LTBI[tw] OR DRTB[tw] OR "DR-TB"[tw] OR XDRTB[tw] OR "XDR-TB"[tw] OR MDRTB[tw] OR "MDR-TB"[tw] OR "Mycobacterium bovis"[tw] OR "M. bovis"[tw] OR "Mycobacterium avium"[tw] OR "M. avium"[tw]

#4 MERGE

#2 AND #3

#5 ADD

Tuberculosis/diagnosis[Mesh]

#6 MERGE

#4 OR #5

FINAL STRATEGY

#1 AND #2 AND #6

2) Global Index Medicus

Same search strategy as for Pubmed

#1 Prison settings

tw:((mh:(prisons)) OR (mh:(prisoners)) OR (tw:(prison*)) OR (tw:(penal)) OR (tw:(jail*)) OR (tw:(reformator*)) OR (tw:(custodial)) OR (tw:(custody)) OR (tw:(gaol*)) OR (tw:(remand*)) OR (tw:(penitentiar*)) OR (tw:(detention*)) OR (tw:(correctional)) OR (tw:(detainee*)) OR (tw:(inmate*)) OR (tw:(imprison*)) OR (tw:(confinement)) OR (tw:(incarcerat*)) OR (tw:(cellmate*)) OR (tw:(lockup*)) OR (tw:(penal institution*)) OR (tw:(detention facilit*)) OR (tw:(bastille*)) OR (tw:(clink*)) OR (tw:(dungeon*)) OR (tw:(guardhouse*)) OR (tw:(slammer*)) OR (tw:(stockade*)) OR (tw:(statesville*)) OR (tw:(up the river)) OR (tw:(brig*)) OR (tw:(bullpen*)) OR (tw:(jailhouse*)) OR (tw:(rack*)) OR (tw:(solitar*)) OR (tw:(stir*)) OR (tw:(black hole*)) OR (tw:(detention camp*)) OR (tw:(house of correction)) OR (tw:(houses of correction)))

#2 Active case finding

tw:((mh:(mass screening)) OR (mh:(mandatory testing)) OR (tw:(screen*)) OR (tw:("case finding")) OR (tw:("case-finding")) OR (tw:(casefinding)) OR (tw:("cases finding")) OR (tw:("case identification")) OR (tw:("cases identification")) OR (tw:(testing)) OR (tw:("rapid test")) OR (tw:("rapid tests")) OR (mh:("early diagnosis")) OR (tw:(early diagnos*)) OR (tw:(early detect*)) OR (tw:(early test*)) OR (tw:("clinical evaluation")) OR (tw:("clinical evaluations")))

#3 TB

tw:((mh:(tuberculosis)) OR (mh:("Mycobacterium tuberculosis")) OR (mh:("Mycobacterium avium")) OR (mh:("Mycobacterium bovis")) OR (tw:(tuberc*)) OR (tw:("Kochs Disease")) OR (tw:("Koch's Disease")) OR (tw:("Koch Disease")) OR (tw:(tb)) OR (tw:(ltb)) OR (tw:(ltbi)) OR (tw:(drtb)) OR (tw:("dr-tb")) OR (tw:(xdrtb)) OR (tw:("xdr-tb")) OR (tw:(mdrtb)) OR (tw:("mdr-tb")) OR (tw:("Mycobacterium bovis")) OR (tw:("m. bovis")) OR (tw:("Mycobacterium avium")) OR (tw:("m. avium")))

#4 MERGE

#2 AND #3

tw:((tw:(w:((mh:(mass screening)) OR (mh:(mandatory testing)) OR (tw:(screen*)) OR (tw:("case finding")) OR (tw:("case-finding")) OR (tw:(casefinding)) OR (tw:("cases finding")) OR (tw:("case identification")) OR (tw:("cases identification")) OR (tw:(testing)) OR (tw:("rapid test")) OR (tw:("rapid tests")) OR (mh:("early diagnosis")) OR (tw:(early diagnos*)) OR (tw:(early detect*)) OR (tw:(early test*)) OR (tw:("clinical evaluation")) OR (tw:("clinical evaluations"))))) AND (tw:(tw:((mh:(tuberculosis)) OR (mh:("Mycobacterium tuberculosis")) OR (mh:("Mycobacterium avium")) OR (mh:("Mycobacterium bovis")) OR (tw:(tuberc*)) OR (tw:("Kochs Disease")) OR (tw:("Koch's Disease")) OR (tw:("Koch Disease")) OR (tw:(tb)) OR (tw:(ltb)) OR (tw:(ltbi)) OR (tw:(drtb)) OR (tw:("dr-tb")) OR (tw:(xdrtb)) OR (tw:("xdr-tb")) OR (tw:(mdrtb)) OR (tw:("mdr-tb")) OR (tw:("Mycobacterium bovis")) OR (tw:("m. bovis")) OR (tw:("Mycobacterium avium")) OR (tw:("m. Avium"))))))

#5 ADD

tw:((mh:(tuberculosis)) AND (mh:(diagnosis)))

#6 MERGE

#4 OR #5

tw:((tw:(tw:((tw:(w:((mh:(mass screening)) OR (mh:(mandatory testing)) OR (tw:(screen*)) OR (tw:("case finding")) OR (tw:("case-finding")) OR (tw:(casefinding)) OR (tw:("cases finding")) OR (tw:("case identification")) OR (tw:("cases identification")) OR (tw:(testing)) OR (tw:("rapid test")) OR (tw:("rapid tests")) OR (mh:("early diagnosis")) OR (tw:(early diagnos*)) OR (tw:(early detect*)) OR (tw:(early test*)) OR (tw:("clinical evaluation")) OR (tw:("clinical evaluations"))))) AND (tw:(tw:((mh:(tuberculosis)) OR (mh:("Mycobacterium tuberculosis")) OR (mh:("Mycobacterium avium")) OR (mh:("Mycobacterium bovis")) OR (tw:(tuberc*)) OR (tw:("Kochs Disease")) OR (tw:("Koch's Disease")) OR (tw:("Koch Disease")) OR (tw:(tb)) OR (tw:(ltb)) OR (tw:(ltbi)) OR (tw:(drtb)) OR (tw:("dr-tb")) OR (tw:(xdrtb)) OR (tw:("xdr-tb")) OR (tw:(mdrtb)) OR (tw:("mdr-tb")) OR (tw:("Mycobacterium bovis")) OR (tw:("m. bovis")) OR (tw:("Mycobacterium avium")) OR (tw:("m. avium")))))))) OR (tw:(tw:((mh:(tuberculosis)) AND (mh:(diagnosis))))))

FINAL STRATEGY

#1 AND #2 AND #6

tw:((tw:(tw:((mh:(prisons)) OR (mh:(prisoners)) OR (tw:(prison*)) OR (tw:(penal)) OR (tw:(jail*)) OR (tw:(reformator*)) OR (tw:(custodial)) OR (tw:(custody)) OR (tw:(gaol*)) OR (tw:(remand*)) OR (tw:(penitentiar*)) OR (tw:(detention*)) OR (tw:(correctional)) OR (tw:(detainee*)) OR (tw:(inmate*)) OR (tw:(imprison*)) OR (tw:(confinement)) OR (tw:(incarcerat*)) OR (tw:(cellmate*)) OR (tw:(lockup*)) OR (tw:(penal institution*)) OR (tw:(detention facilit*)) OR (tw:(bastille*)) OR (tw:(clink*)) OR (tw:(dungeon*)) OR (tw:(guardhouse*)) OR (tw:(slammer*)) OR (tw:(stockade*)) OR (tw:(statesville*)) OR (tw:(up the river)) OR (tw:(brig*)) OR (tw:(bullpen*)) OR (tw:(jailhouse*)) OR (tw:(rack*)) OR (tw:(solitar*)) OR (tw:(stir*)) OR (tw:(black hole*)) OR (tw:(detention camp*)) OR (tw:(house of correction)) OR (tw:(houses of correction))))) AND (tw:(tw:((mh:(mass screening)) OR (mh:(mandatory testing)) OR (tw:(screen*)) OR (tw:("case finding")) OR (tw:("case-finding")) OR (tw:(casefinding)) OR (tw:("cases finding")) OR (tw:("case identification")) OR (tw:("cases identification")) OR (tw:(testing)) OR (tw:("rapid test")) OR (tw:("rapid tests")) OR (mh:("early diagnosis")) OR (tw:(early diagnos*)) OR (tw:(early detect*)) OR (tw:(early test*)) OR (tw:("clinical evaluation")) OR (tw:("clinical evaluations"))))) AND (tw:(tw:((tw:(tw:((tw:(w:((mh:(mass screening)) OR (mh:(mandatory testing)) OR (tw:(screen*)) OR (tw:("case finding")) OR (tw:("case-finding")) OR (tw:(casefinding)) OR (tw:("cases finding")) OR (tw:("case identification")) OR (tw:("cases identification")) OR (tw:(testing)) OR (tw:("rapid test")) OR (tw:("rapid tests")) OR (mh:("early diagnosis")) OR (tw:(early diagnos*)) OR (tw:(early detect*)) OR (tw:(early test*)) OR (tw:("clinical evaluation")) OR (tw:("clinical evaluations"))))) AND (tw:(tw:((mh:(tuberculosis)) OR (mh:("Mycobacterium tuberculosis")) OR (mh:("Mycobacterium avium")) OR (mh:("Mycobacterium bovis")) OR (tw:(tuberc*)) OR (tw:("Kochs Disease")) OR (tw:("Koch's Disease")) OR (tw:("Koch Disease")) OR (tw:(tb)) OR (tw:(ltb)) OR (tw:(ltbi)) OR (tw:(drtb)) OR (tw:("dr-tb")) OR (tw:(xdrtb)) OR (tw:("xdr-tb")) OR (tw:(mdrtb)) OR (tw:("mdr-tb")) OR (tw:("Mycobacterium bovis")) OR (tw:("m. bovis")) OR (tw:("Mycobacterium avium")) OR (tw:("m. avium")))))))) OR (tw:(tw:((mh:(tuberculosis)) AND (mh:(diagnosis)))))))))

3) Cochrane Library

#1 Prison settings

MeSH descriptor: [prisons] explode all trees OR MeSH descriptor: [prisoners] explode all trees OR prison*:ti,ab,kw OR penal*:ti,ab,kw OR jail*:ti,ab,kw OR reformator*:ti,ab,kw OR custodial:ti,ab,kw OR custody:ti,ab,kw OR gaol*:ti,ab,kw OR remand*:ti,ab,kw OR penitentiar*:ti,ab,kw OR detention*:ti,ab,kw OR correctional:ti,ab,kw OR detainee*:ti,ab,kw OR inmate*:ti,ab,kw OR imprison*:ti,ab,kw OR confinement:ti,ab,kw OR incarcerat*:ti,ab,kw OR cellmate*:ti,ab,kw OR lockup*:ti,ab,kw OR penal institution*:ti,ab,kw OR detention facilit*:ti,ab,kw OR bastille*:ti,ab,kw OR clink*:ti,ab,kw OR dungeon*:ti,ab,kw OR guardhouse*:ti,ab,kw OR slammer*:ti,ab,kw OR stockade*:ti,ab,kw OR statesville*:ti,ab,kw OR up the river:ti,ab,kw OR brig*:ti,ab,kw OR bullpen*:ti,ab,kw OR jailhouse*:ti,ab,kw OR rack*:ti,ab,kw OR solitar*:ti,ab,kw OR stir*:ti,ab,kw OR black hole*:ti,ab,kw OR detention camp*:ti,ab,kw OR house of correction:ti,ab,kw OR houses of correction:ti,ab,kw

The final strategy will exclusively use search string #1. If the number of references found is too high for timely screening, search strings for TB and screening will be applied in the same ways as the PubMed and Global Index Medicus databases but adapted to the Cochrane Library database's requirements.

Appendix 2. The QUADAS-2 Tool.

This review reuses the signaling questions applied by van't Hoog *et al.* in their review protocol "Symptom- and chest-radiography screening for active pulmonary tuberculosis in HIV-negative adults and adults with unknown HIV status" [19].

Table. Signaling questions for risk of bias assessment [19].

Key questions	Signaling questions
**Domain 1: Patient selection**	
Risk of bias: Could the selection of patients have introduced bias?	1. Did the study enroll a consecutive or random sample of patients?<br>• Yes: if all eligible patients were enrolled; or if the authors reported that the patients were either a consecutive series or randomly selected;<br>• No: if the authors report that the selection was based on clinical judgment of health workers, or participation of randomly selected people in the study was low;<br>• Unclear: if there is a discrepancy between the numbers of eligible people and the number of included people, but no reasons are given for that, or the selection procedure is not clearly described.<br><br>2. Was a case-control design avoided?<br>• Yes: if a case-control design was avoided;<br>• No: if a case-control design was not avoided;<br>• Unclear: if not reported or insufficient information is provided to decide.<br><br>3. Did the study avoid inappropriate exclusions?<br>• Yes: if no study participants were excluded after inclusion;<br>• No: if study participants were excluded (for example, participants with mild or severe symptoms or signs);<br>• Unclear: if insufficient information is provided to decide.
Applicability: Are there concerns that the included patients and setting do not match the review question?	• High concern: if the study population does not resemble a population that would be considered for a TB screening program in practice;<br>• Low concern: if the study population does resemble a population that would be considered for a TB screening program in practice;<br>• Unclear: if not reported or insufficient information is provided to decide.
**Domain 2: Index test**	
Risk of bias: Could the conduct or interpretation of the index test have introduced bias?	1. Were the index test results interpreted without knowledge of the results of the reference standard?<br>• Yes: if the screening test was performed without knowing whether the person had infectious TB;<br>• No: if symptom questions were asked after the reference test results were known, or the CXR was interpreted with knowledge of the results of the reference test;<br>• Unclear: if insufficient information is provided to decide (for example, if it was unclear whether the CXR reader was blinded to the reference test results).<br><br>2. If a threshold was used, was it pre-specified?<br>• This question did not apply to our review question.
**Domain 3: Reference standard**	
Risk of bias: Could the reference standard, its conduct, or its interpretation have introduced bias?	1. Is the reference standard likely to classify the target condition correctly?<br>• Yes: if the reference standard was an author-defined combination of mycobacterial culture (on solid or liquid medium) and possibly sputum smear microscopy, or Xpert, or both, and cases defined by sputum microscopy only are limited to a small proportion (≤10%) in whom culture was contaminated or negative or missing, but smears were positive;<br>• No: if the reference standard was not an author-defined combination of mycobacterial culture (on solid or liquid medium) and possibly sputum smear microscopy, or Xpert, or both. That includes studies where sputum smear microscopy was the only reference test;<br>• Unclear: if insufficient information is provided to decide.<br><br>2. Were the reference standard results interpreted without knowledge of the results of the index test?<br>• Yes: if the screening test results were not known to the people interpreting the reference standard results;<br>• No: if the screening test results were known to the people interpreting the reference standard results;<br>• Unclear: if insufficient information is provided to decide.
Applicability: Are there concerns that the target condition as defined by the reference standard does not match the question?	• High concern: if there was a high probability that a considerable proportion of the TB cases identified in the study did not have bacteriologically confirmed TB or did not have active TB;<br>• Low concern: (i) if the TB cases in the study have TB symptoms or CXR abnormalities in addition to a positive culture, or positive smear microscopy, or both; or (ii) if they have at least two different samples positive on culture, or smear microscopy, or both;<br>• Moderate concern: Because we perceive a large contrast between "low" and "high," we added a category "moderate" for the applicability sections. We applied the "moderate" category if the TB cases in the study could include people with one positive sputum culture or Xpert, NAAT, or smear only, without the presence of symptoms or CXR abnormalities;<br>• Unclear: if insufficient information is provided to decide.

Table. Continued.

Key questions	Signaling questions
**Domain 4: Flow and timing**	
Risk of bias: Could the patient flow have introduced bias?	1. Was there an appropriate interval between the index test and reference standard?<br>• Yes: if the screening test and reference standard were applied (or samples were taken) at the same time or within one week;<br>• No: if the time between the screening test and reference standard (sample collection) was more than one week;<br>• Unclear: if insufficient information is provided to decide.<br><br>2. Did all patients receive the same reference standard?<br>• Yes: if all participants were evaluated with the reference standard, and if all or a large majority of participants were evaluated with the same test(s);<br>• No: if not all participants were evaluated with the reference;<br>• standard, or participants received different tests (for example, some smear only, some culture, or different numbers of samples were submitted for testing);<br>• Unclear: if insufficient information is provided to decide.<br><br>3. Were all patients included in the analysis?<br>• Yes: if all participants were included;<br>• No: if participants who participated were excluded. For instance, because they did not provide sputum for a reference test;<br>• Unclear: if insufficient information is provided.

CXR – Chest radiography; NAAT – Nucleic acid amplification test; TB – Tuberculosis.

The guiding criteria for review assessment of the quality of diagnostic accuracy of active PTB screening algorithms were developed for use with the QUADAS 2 tool [29]. The category "unclear" should be assigned only when there is general uncertainty in scoring each item. Review authors should contact the study team for more information in these cases.
